# Predicting neurological outcome after out-of-hospital cardiac arrest with cumulative information; development and internal validation of an artificial neural network algorithm

**DOI:** 10.1186/s13054-021-03505-9

**Published:** 2021-02-25

**Authors:** Peder Andersson, Jesper Johnsson, Ola Björnsson, Tobias Cronberg, Christian Hassager, Henrik Zetterberg, Pascal Stammet, Johan Undén, Jesper Kjaergaard, Hans Friberg, Kaj Blennow, Gisela Lilja, Matt P. Wise, Josef Dankiewicz, Niklas Nielsen, Attila Frigyesi

**Affiliations:** 1 Department of Clinical Sciences Lund, Anaesthesia and Intensive Care, Lund University, Skåne University Hospital, Lund, Sweden; 2 Department of Clinical Sciences Lund, Anesthesia and Intensive Care, Lund University, Helsingborg Hospital, Lund, Sweden; 3 Department of Energy Sciences, Faculty of Engineering, Lund University, Lund, Sweden; 4 Centre for Mathematical Sciences, Mathematical Statistics, Lund University, Lund, Sweden; 5 Department of Clinical Sciences Lund, Neurology, Lund University, Skåne University Hospital, Lund, Sweden; 6 Department of Cardiology, Rigshospitalet and Department of Clinical Medicine, University of Copenhagen, Copenhagen, Denmark; 7 Department of Psychiatry and Neurochemistry, Institute of Neuroscience and Physiology, The Sahlgrenska Academy At the University of Gothenburg, Mölndal, Sweden; 8 Clinical Neurochemistry Laboratory, Sahlgrenska University Hospital, Mölndal, Sweden; 9 Department of Neurodegenerative Disease, UCL Institute of Neurology, Queen Square, London, UK; 10 UK Dementia Research Institute At UCL, London, UK; 11 Medical and Health Directorate, National Fire and Rescue Corps, 1, rue Robert Stumper, 2557 Luxembourg, Luxembourg; 12 Department of Clinical Sciences Malmö, Anaesthesia and Intensive Care, Lund University, Hallands Hospital Halmstad, Halland, Sweden; 13 Department of Clinical Sciences Lund, Anaesthesia and Intensive Care, Lund University, Skåne University Hospital, Malmö, Sweden; 14 Adult Critical Care, University Hospital of Wales, Cardiff, UK; 15 Department of Clinical Sciences Lund, Cardiology, Lund University, Skåne University Hospital, Lund, Sweden; 16 Department of Intensive and Perioperative Care, Skåne University Hospital, Getingevägen 4, 222 41 LundLund, Sweden

**Keywords:** Machine learning, Artificial intelligence, Artificial neural networks, Neural networks, Out-of-hospital cardiac arrest, Cardiac arrest, Cerebral performance category, Critical care, Intensive care, Prediction, Prognostication

## Abstract

**Background:**

Prognostication of neurological outcome in patients who remain comatose after cardiac arrest resuscitation is complex. Clinical variables, as well as biomarkers of brain injury, cardiac injury, and systemic inflammation, all yield some prognostic value. We hypothesised that cumulative information obtained during the first three days of intensive care could produce a reliable model for predicting neurological outcome following out-of-hospital cardiac arrest (OHCA) using artificial neural network (ANN) with and without biomarkers.

**Methods:**

We performed a post hoc analysis of 932 patients from the Target Temperature Management trial. We focused on comatose patients at 24, 48, and 72 h post-cardiac arrest and excluded patients who were awake or deceased at these time points. 80% of the patients were allocated for model development (training set) and 20% for internal validation (test set). To investigate the prognostic potential of different levels of biomarkers (clinically available and research-grade), patients’ background information, and intensive care observation and treatment, we created three models for each time point: (1) clinical variables, (2) adding clinically accessible biomarkers, e.g., neuron-specific enolase (NSE) and (3) adding research-grade biomarkers, e.g., neurofilament light (NFL). Patient outcome was the dichotomised Cerebral Performance Category (CPC) at six months; a good outcome was defined as CPC 1–2 whilst a poor outcome was defined as CPC 3–5. The area under the receiver operating characteristic curve (AUROC) was calculated for all test sets.

**Results:**

AUROC remained below 90% when using only clinical variables throughout the first three days in the ICU. Adding clinically accessible biomarkers such as NSE, AUROC increased from 82 to 94% (*p* < 0.01). The prognostic accuracy remained excellent from day 1 to day 3 with an AUROC at approximately 95% when adding research-grade biomarkers. The models which included NSE after 72 h and NFL on any of the three days had a low risk of false-positive predictions while retaining a low number of false-negative predictions.

**Conclusions:**

In this exploratory study, ANNs provided good to excellent prognostic accuracy in predicting neurological outcome in comatose patients post OHCA. The models which included NSE after 72 h and NFL on all days showed promising prognostic performance.

**Supplementary Information:**

The online version contains supplementary material available at 10.1186/s13054-021-03505-9.

## Introduction

To estimate the prognosis for long-term neurological recovery in patients who remain comatose during the first few days after resuscitation from a cardiac arrest is a common and important part of intensive care. Patients’ background, cardiac arrest characteristics, vital signs on hospital admission, and findings from diagnostic investigations are all contributing factors which make prognostication complex [1]. There is a need for robust and reliable methods to analyse data and assist in prognostication where a full recovery or severe neurological deficits are possible long-term clinical outcomes.

The prognostication process should, according to the latest guidelines, be deferred for at least 72 h after the return of spontaneous circulation (ROSC) and should be multimodal [2, 3]. The clinical neurological examination is the foundation of this process and is supported by electroencephalography (EEG), somatosensory-evoked potentials (SSEP), neuroradiological imaging, and one biomarker. Although demographic and clinical variables carry important prognostic information, none are included in current algorithms [4].

In recent years, a number of biomarkers have emerged, which potentially could improve current algorithms for the prediction of neurological outcome. Today, only neuron-specific enolase (NSE) is included in the guidelines [4]. Amongst many novel biomarkers that have been studied for brain injury after cardiac arrest, the most promising so far is neurofilament light (NFL), with an area under the receiver operating characteristic curve (AUROC) of 94–98% for discrimination of long-term neurological outcome as early as 24 h after ROSC [5, 6]. Other biomarkers of brain injury, including S100 calcium-binding protein B (S100B), tau protein, glial fibrillary acidic protein (GFAP), and ubiquitin C-terminal hydrolase-L1 (UCHL1), have also shown potential in cardiac arrest prognostication [7–11]. Biomarkers of cardiac injury such as troponin T (TnT), N-terminal pro–B-type natriuretic peptide (BNP) and copeptin along with biomarkers of systemic inflammation such as procalcitonin (PCT) and interleukin-6 (IL-6) are also associated with neurological outcome [12–16]. Some of these biomarkers are routinely measured in many laboratories, while others are not. Despite substantial efforts to determine promising biomarkers, the prognostic value of combining and adding them to cumulative clinical data remains unclear [12].

Improvements in machine learning algorithms and increased computational power have led to an enhanced diagnostic and prognostic capability in a variety of medical fields, ranging from radiology to intensive care medicine [17, 18]. Machine learning has also shown promising results in short-term and long-term prognostication in survivors of out-of-hospital cardiac arrest (OHCA) [19, 20]. In a recently published study, we showed how a supervised machine learning algorithm called artificial neural networks (ANN) was superior to logistic regression when predicting long-term neurological outcome including survival, based on information available on hospital admission [20].

In this extension of our previous study, we hypothesised that cumulative information obtained during the first three days of intensive care could produce a reliable model for predicting neurological outcome post OHCA using ANN with and without biomarkers.

## Materials and methods

### Study population and variables

We included all 939 patients from the primary analysis of the Target Temperature Management (TTM) trial [21], which randomised unconscious OHCA survivors to compare two target temperatures of 33 °C and 36 °C upon ICU admission. Patients from 36 ICUs across Europe and Australia were enrolled between 2010 and 2013. The inclusion criteria were comatose (Glasgow Coma Scale (GCS) ≤ 8) adults (≥ 18 years of age) with a sustained ROSC after resuscitation from OHCA of presumed cardiac cause. The primary outcome was mortality until the end of the trial, which did not differ significantly between the temperature groups [21]. The trial protocol was approved by the ethical committees in each participating country, and informed consent was either waived or obtained from all participants or relatives according to the national legislation, in line with the Helsinki declaration [22, 23].

**Fig. 1:**
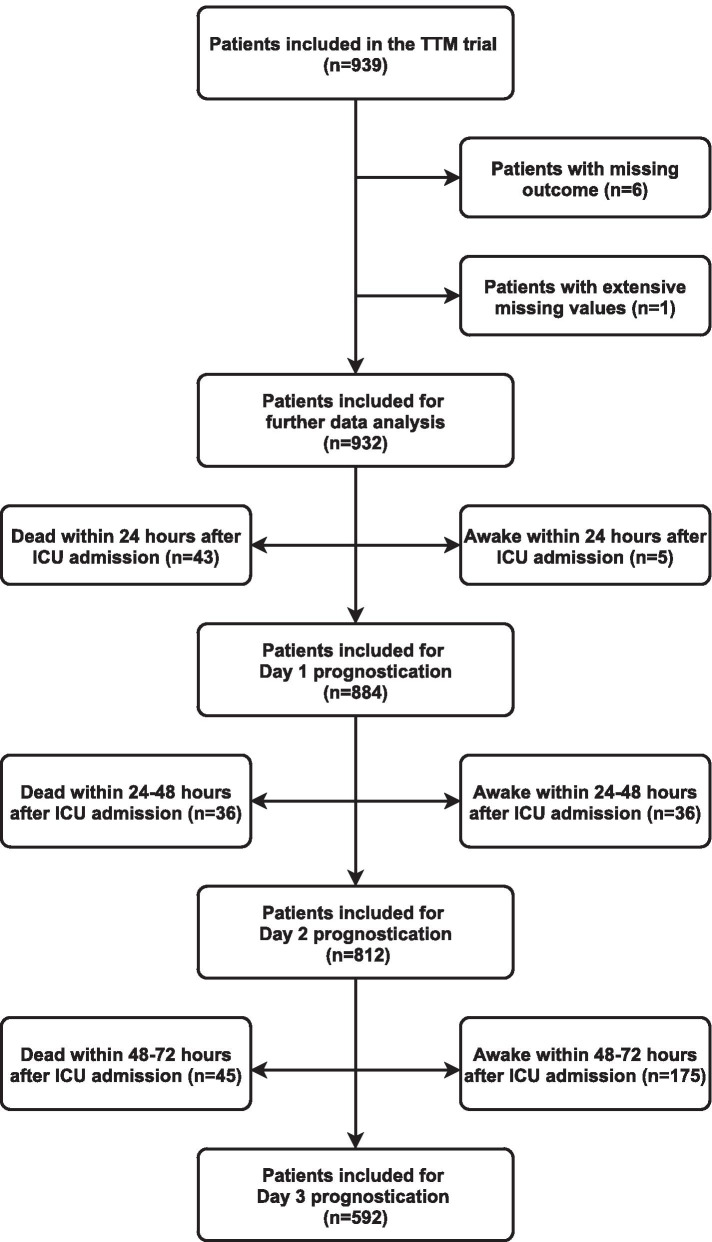
Flowchart. Flowchart for the study populations at day 1, 2, and 3 after admission to ICU. Population characteristics in Table 1 are based on ‘Patients included for further data analysis (*n* = 932)’. TTM, target temperature management. ICU, intensive care unit

Patients without follow-up at six months or an extensively high number of missing values (> 40 missing values on hospital admission) were excluded (*n* = 7) from the final data analysis. We studied three different time points after cardiac arrest (24, 48 and 72 hours). To limit prognostication to comatose patients, we excluded patients who were awake or deceased at these times (Fig. 1). Awake was defined as GCS motor response score of 6 (measured on a daily basis), where the patient obeys commands for movement or Cerebral Performance Category (CPC) 1–3 at ICU discharge.

All variables from the TTM-trial up to day 3 were included; background information, prehospital and hospital admission records along with data obtained at 24 h (day 1), 48 h (day 2), 72 h (day 3). All variables are displayed in Additional file 1: table s1A; Additional file 2: table s1b; Additional file 3: table s1c; Additional file 4: table s1D in the electronic supplement. Computed tomography (CT), magnetic resonance imaging (MRI), EEG and SSEP were not included as these modalities were used in a minority of patients.

The TTM-trial biobank collected blood samples from 29 of the 36 trial sites on day 1,2 and 3 and comprised approximately 70% of the total TTM-trial patient population. Biomarkers analysed in the biobank were grouped by whether they were considered clinically accessible or research-grade. Three models (A, B and C) were developed for each of the three days studied (a total of nine datasets):

- Level A: Clinical variables only
- Level B: A & clinically accessible biomarkers: NSE, S100B, TnT, BNP, and PCT
- Level C: B & research-grade biomarkers: NFL, copeptin, IL-6, tau, GFAP, and UCHL1

To ensure that the prognostic value of the biomarkers would not be weakened by the imputation technique, we excluded patients with missing values corresponding to the exact day the data was missing for NSE and NFL in level B and C, respectively, which resulted in level B and C having approximately 30% fewer patients in each dataset.

### Outcome

The outcome was a dichotomised CPC scale graded by a blinded assessor after an interview face-to-face or by telephone at the six-month follow-up [24]. A good outcome was defined as CPC 1–2 and poor outcome as CPC 3–5. A good outcome means independence in activities of daily living but may include minor disability. A poor outcome means severe brain injury; dependence on others, coma or death [25].

### Model development

To ensure an unbiased model development and independent internal validation, 80% of the patients were randomly allocated to development (training set), and 20% were allocated to validation (test set). The training/test split remained constant throughout the model development. We aimed to create prediction models for long-term neurological outcome based on background, prehospital, and hospital admission data along with available ICU information obtained on day 1, 2 and 3 after admission based on the following levels: A) without biomarkers, B) adding clinically accessible biomarkers and C) adding research-grade biomarkers.

For each model, we used the following plan for variable selection and missing value imputation:

- A maximum of 20% missing values for each variable,
- A minimum of 2% unique events for binary variables,
- A maximum of 98% correlation between variables,
- Median or mode imputation to handle missing values below the 20% threshold.
- To reduce the number of variables in each model, we used a wrapper variable selection which combines both the Boruta variable selection and Shapley values [26].

We used an ANN to predict the neurological outcome at six months. An ANN consists of an input layer, a number of hidden layers and an output layer. These layers consist of nodes which can aggregate information from previous layers, transform it, and then send it forward to the next layer. The aim is to mimic the complex network of connected neurons in the human nervous system and thereby detect patterns and dependencies between variables and outcome to improve prognostic performance. We used fivefold cross-validation and a Bayesian optimisation algorithm for hyperparameter tuning during development to find the best possible model within the following constraints: 1–3 hidden layers, 5–250 hidden nodes in each layer, batch size between 4 and 128, a drop-out rate between 0 and 0.5 for the input layer and 0–0.5 for the hidden layers, a fixed learning rate of 10^–3^, and the activation function for the hidden layers was chosen to be either the rectified linear unit (ReLU) or the hyperbolic tangent function. We used either L_1_, L_2_ or max-norm for norm-regularisation. All networks were trained using early stopping with a patience of 30 epochs and a maximum number of 1000 epochs. We used binary cross entropy as our loss function. The sigmoid activation function was used for the output layer. We chose the model with the highest mean AUROC of the cross-validations for further analysis.

After the model development, we applied Shapley additive explanation algorithm (SHAP) to all models to visualise which variables explained an individual prediction and to understand the relative contribution of variables. The SHAP algorithm is based on Shapley values, which originate from cooperative game theory, and explains how much a single variable contributes to the difference between the actual prediction and the mean of all predictions. The SHAP algorithm can help explain how a prediction model works and mitigate some of the concerns about "black box" modelling. We created one patient example to illustrate the explanation of a patient-specific prediction on day 1–3 (level C). For all nine models, we calculated the mean of the absolute SHAP values for each variable and displayed it using a bar plot to rank the variables for each model.

### Statistical analysis methods

All continuous variables are presented as medians with interquartile ranges (IQR). Categorical variables are presented as numbers and percentages. Missing data are presented as percentages. The Mann–Whitney U test was used for comparison between groups of continuous data, and Fisher's exact test was used for categorical data. We evaluated the prediction models using the receiver operating characteristic (ROC) curve and calculated the AUROC for all models based on test sets. To evaluate the prognostic capability of our models, we calculated a confusion matrix for all test sets, based on the threshold for 100% specificity in the corresponding training set to display; True-positive (TP), true-negative (TN), false-positive (FP) and false-negative (FN) predictions. All p-values were two-tailed, and *p* < 0.05 were considered significant.

Statistical analyses were done in R (The R Foundation for Statistical Computing) and Python [27, 28]. All ANN models were created using Tensorflow 2.0, an open-source framework developed by Google [29]. The 'Boruta-Shap' Python package was used for variable selection [26]. The post-hoc explanation of the ANN models was based on the 'shap' package in Python [30]. We used the 'pROC' and 'Optimalcutpoints’ package in R when producing the ROC curves and calculating the threshold for the confusion matrix [31, 32]. The TRIPOD statement was followed when writing this manuscript [33].

## Results

We included 932 patients from the TTM-trial after excluding six patients due to missing outcomes and one patient due to missing values. Overall poor outcome (CPC 3–5) was found in 492 (53%) patients while good outcome (CPC 1–2) was found in 440 (47%) patients [20]. The population characteristics are shown in Additional file 1: table s1A; Additional file 2: table s1b; Additional file 3: table s1c; Additional file 4: table s1D (Supplement), which includes patients’ background, prehospital and admission characteristics, standard ICU observations and treatment along with biomarkers obtained on day 1, 2, and 3. As shown in Fig. 1, we excluded patients who were deceased or woke up by 24 h, between 24 and 48 h, and between 48 and 72 h in our analysis of day 1, 2 and 3, respectively. As described in the methods section, three datasets were then created for each day based on the level of additional biomarkers. The number of patients in each dataset along with the number of variables are shown in Table 1.

**Table 1:** Overview and prognostic performance

Timeline	Type of data	Number of patients			Number of variables		Model performance		Confusion matrix (test set)				
		Total (n)	Train set (n)	Test set (n)	Before variable selection (n)	After variable selection (n)	Training set (cross validation) AUROC (%) (CI 95%)	Test set AUROC (%) (CI 95%)	Probability threshold	TN (n)	FP (n)	FN (n)	TP (n)
Day 1 (24 h)	Level A	884	702	182	120	22	85.7 (83.2–88.6)	81.9 (75.9–87.9)	0.981	99	0	82	1
	Level B	638	502	136	125	21	89.9 (86.8–92.2)	81.8 (74.9–88.6)	0.983	70	0	57	9
	Level C	690	545	145	131	22	96.6 (94.7–97.5)	95.2 (91.9–98.4)	0.887	76	1	21	47
Day 2 (48 h)	Level A	812	645	167	174	21	85.8 (82.9–88.5)	78.1 (71.1–85.0)	0.935	88	2	70	7
	Level B	578	460	118	184	17	93.7 (91.7–95.9)	89.7 (84.3–95.0)	0.963	59	0	44	15
	Level C	624	495	129	196	21	96.6 (95.2–97.9)	96.1 (93.4–98.8)	0.986	67	0	31	31
Day 3 (72 h)	Level A	592	469	107	228	17	84.9 (80.6–87.9)	86.7 (80.4–93.1)	0.920	60	0	22	25
	Level B	415	328	87	243	12	92.7 (89.8–95.1)	94.1 (89.4–98.8)	0.885	40	0	22	25
	Level C	442	347	95	261	23	96.6 (94.9–98.1)	94.7 (90.6–98.7)	0.820	45	0	16	34

Overview and prognostic performance of the ANN models during the first three days after ICU admission. In level A, we used all available data from the TTM-trial, in level B, we added clinically accessible biomarkers, and for level C we added research-grade biomarkers as well. The prognostic performance is displayed as the area under the receiver operating characteristic curve (AUROC) and using by a confusion matrix. Note the threshold for the confusion matrix was based on the threshold for 100% specificity in the training set. TN, true negative; TP, true positive; FN, false negative; TP, true positive

**Fig. 2:**
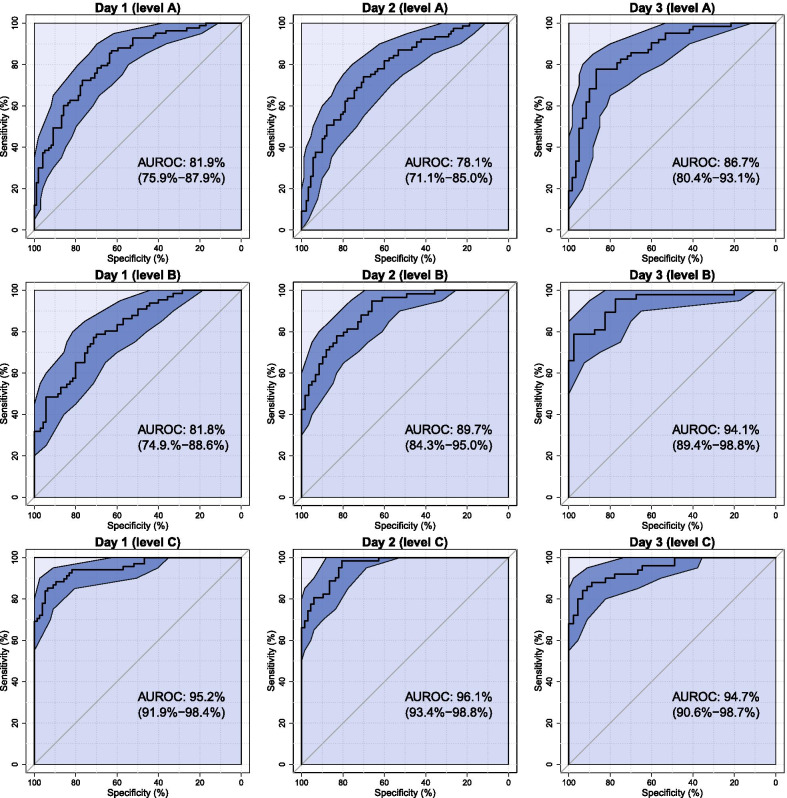
Prognostic performance. Receiver operating characteristic curves (ROC) and areas under the curves (AUROC) for all nine models including 95% confidence intervals. The figures present the capability of the models to discriminate patients with poor outcome (Cerebral Performance Category [CPC] 3–5) and those with good outcome (CPC 1–2) at six-months on an independent test set. The rows represent the different levels of added biomarkers to the available standard intensive care unit (ICU) observations from the TTM-trial: None (level A), clinically accessible biomarkers (level B), and research-grade biomarkers (level C). The columns represent the timeline after admission to the ICU. For each ROC curve, the 95% CI was calculated for specificity at different levels of sensitivity and displayed as a blue 95 CI band. TTM, target temperature management. CI, confidence interval

All models from day 1 to day 3 showed good to excellent prognostic performance in predicting neurological outcome at six months (see Fig. 2). Using clinical variables (level A), the AUROC remained under 90% throughout the first three days of intensive care. Upon adding the clinically accessible biomarkers (level B), the AUROC increased from 82 to 94% (*p* < 0.01). For the model with research-grade biomarkers (level C), the prognostic performance was excellent from day 1 to day 3 with an AUROC at approximately 95% (see Table 1 and Fig. 2). In summary, adding clinically accessible biomarkers to the clinical variables in level B successively improved the prognostication, whereas levels A and C both showed similar results on all days. Furthermore, as seen in Fig. 2, the sensitivity was above 60% while retaining a specificity of 100% for level B at 72 h and level C at all time points.

Based on a threshold with 100% specificity (no false positives) for patients in the training set, false-positive predictions (predicted poor outcome, reported good outcome) occurred in two models in the test set; 0.7% (day 1 level C) and 1.2% (day 2 level A). The rate of false-negative predictions (predicted good outcome, reported poor outcome) was high for the majority of the models but remained below 25% throughout day 1–3 when the research-grade biomarkers were included (level C) (see Table 1).

**Fig. 3:**
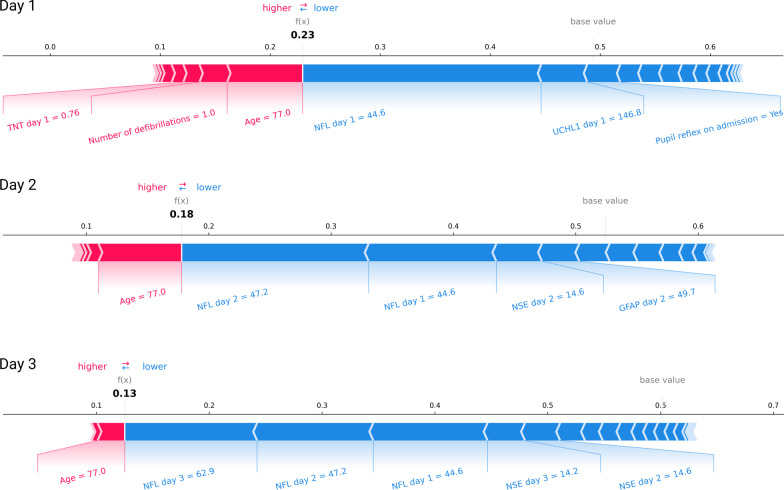
Illustration of the impact of features for a patient-specific prediction. An example of patient-specific prediction using the Shapley additive explanations algorithm (SHAP). This patient is predicted to have a 23% risk of a poor outcome on day 1, and 18% and 13% risk of a poor outcome on days 2 and 3, respectively **(**using the level C model). The patient’s age is a is the most important risk-increasing variable while the modest levels of biomarkers like NFL, NSE decrease the risk a poor outcome. TNT, Troponin-T (ng/L). NFL, Neurofilament light (ng/L). UCHL1, Ubiquitin carboxy-terminal hydrolase L1 (ng/L). NSE, Neuron-specific enolase (ng/ml.) GFAP, Glial fibrillary acidic protein (ng/L)

**Fig. 4:**
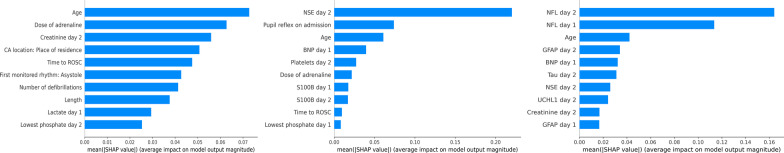
SHAP variable importance on day 2. The global importance of each feature of each model illustrated with the SHAP variable importance. The most important variable has the highest mean of absolute SHAP values. The top ten SHAP variable importance are shown for level A-C on day 2 (after 48 h). The left panel shows level A (without adding biomarkers), the middle panel shows level B (adding clinically accessible biomarkers), and the right panel shows level C (adding research-grade biomarkers). The ranking of the variables depend on which biomarkers were included in the model. For level A (no biomarkers), age and the dose of adrenaline during resuscitation were the variables carrying the most information. In level B and C, age was ranked as the third most important variable when more information was provided by biomarkers. The dose of adrenaline was reduced to the sixth most important variable when adding clinically accessible biomarkers and was not included in the top ten variables when including research-grade biomarkers as well. SHAP, Shapley additive explanations algorithm. CA, cardiac arrest. ROSC, return of spontaneous circulation. NSE, Neuron-specific enolase. BNP, brain natriuretic protein. S100B, S100 calcium-binding protein B. NFL, Neurofilament light. GFAP, Glial fibrillary acidic protein. Tau. UCHL1, Ubiquitin carboxy-terminal hydrolase L1

By applying the SHAP algorithm to our models, the predictions could be explained. This is illustrated in Fig. 3, where the ANN model for level C is used to predict the probability of a poor outcome in a patient where the SHAP algorithm is used to explain the prediction. In this example, the patient’s age of 77 years increases the risk of a poor outcome, whereas the low levels of biomarkers (i.e. NFL and NSE) decrease the risk of a poor outcome. Furthermore, we used the SHAP algorithm to rank the importance of the variables for every model. In Fig. 4, the ten most important variables for level A, B and C on day 2 were ranked by variable importance (see Supplementary for the top ten rankings of variables for day 1 and 3). For level A, age and the dose of adrenaline during resuscitation were the variables carrying the most value. In level B and C, age was ranked as the third most important variable when more information was gained by biomarkers. The dose of adrenaline was reduced to the sixth most important variable and was not included in the top ten variables, when clinically accessible and research-grade biomarkers were added, respectively.

## Discussion

In this exploratory retrospective study, we found that models using clinical variables paired with biomarkers and machine learning appear promising in predicting long-term neurological outcome in comatose patients post cardiac arrest. Using only clinical variables resulted in moderate predictive ability. However, when clinically accessible biomarkers such as NSE were added, the predictive capability improved over time, reaching an AUROC of 94%, which supports the role of NSE in current guidelines. As seen in Fig. 4, age, BNP, and platelets also contributed to the prediction on day 3 for level B. With research-grade biomarkers added, the prognostic capability was excellent with an AUROC of approximately 95% already evident after 24 h post cardiac arrest. Research-grade biomarkers, such as NFL and GFAP, carried the most robust predictive information, and over time they rendered clinical variables largely redundant with little additive value, as supported by our previous paper, showing a high AUC value (94%) of NFL alone [5]. Additional analysis of NFL alone showed excellent prognostic value with an AUROC of 92.3%, 92.3% and 92.5% on day 1,2 and 3, respectively (based on the test set) when using this study design.

The overall objective in cardiac arrest prognostication is to aim for zero false-positive predictions (predicted poor outcome, reported good outcome), which would result in the withdrawal of life-sustaining therapies in a patient who otherwise would have survived with a good outcome. The specificity is, therefore, arguably more important than sensitivity.

The latest treatment guidelines for comatose cardiac arrest survivors recommend multimodal prognostication to be performed at least 72 h after ROSC. Moseby-Knappe et al. found that the current 4-step algorithm for neurological prognostication after cardiac arrest recommended by the European Resuscitation Council (ERC) and the European Society of Intensive Care Medicine (ESICM) identified patients with poor outcome with a 39% sensitivity and 100% specificity [34]. These guidelines are based on the consensus opinion of leading experts in the field. In contrast to that study, the current findings suggest that even at 24 h following cardiac arrest the prognostic performance is excellent with an AUROC of 95% (95% CI: 92–98%) and sensitivity above 60% while retaining 100% specificity upon adding research-grade biomarkers. These results are mainly driven by the biomarker NFL and are similar to two previous studies, of which one is based on the same data (TTM-trial) [5, 6]. In fact, level B on day 3 (72 h) as well as level C for all time points had a sensitivity above 60% while retaining 100% specificity, which is comparable with the performance reported using the ERC/ESICM guidelines, which included important prognostics such as SSEP, EEG and neuroradiological imaging. This is noteworthy, especially for level B on day 3, which is mainly driven by NSE values. Both NSE after 72 h and NFL at all time points have previously shown good to excellent performance individually with a sensitivity of 52% and 53–65% while retaining 100% specificity, respectively [5, 35]. Our study differs from those studies by only including comatose patients.

To further investigate this, we based the prediction of poor outcome in the test set on the threshold of 100% specificity in the training set. This does not guarantee a zero false-positive rate in the test set, as it does not account for the lower bound in the confidence interval or outliers. Nevertheless, the false-positive predictions for the model, including research-grade biomarkers, were less than 1% on day 1, and 0% on days 2 and 3 with a reasonably low false-negative rate of 15–25%. Consequently, the specificity for level C on day 1 was not 100% based on the threshold in the training set. For the model including clinically accessible biomarkers on day 3, the false-positive predictions were 0% and the false-negative rate was also reasonably low (25%).

Deep learning algorithms are new for OHCA prognostication. In a study from 2019, Kwon et al. showed that a deep learning algorithm outperformed other types of supervised machine learning based on a validation set of 8,145 patients when predicting a poor outcome (CPC 3–5) on hospital discharge [19]. They reported an AÙROC of 95% with narrow confidence intervals based on information available at the time of ROSC only. Their study differs from ours as we looked at the six-month neurological outcome and focused on comatose patients admitted to the ICU, where the balance between good and poor outcome is around 50%.

To our knowledge, this is the first prediction study using cumulative data in the first three days of ICU admission and the first to combine the predictive capability of different groups of biomarkers by adding them to clinical variables. The cumulative approach is a natural step after testing biomarkers individually to understand how they are ranked and interact over time. The SHAP algorithm allows for each prediction to be explained, which can help both researchers and physicians in treating OHCA patients and understanding the dynamic between the variables better, both for the individual patient and as a group.

The ranking of the variables depends on which biomarkers were included in the models. As shown in Fig. 4, age and the dose of adrenaline during resuscitation were the variables carrying the most information on day 2 (level A). The reason why the dose of adrenaline plays such an important role could be related to its correlation to other predictors such as time to ROSC and initial cardiac rhythm. The biomarkers dominated the top ten ranked variables upon being added to the models. Age remained an important variable in all models because age itself is a risk factor and possibly due to its correlation with comorbidities.

There are several limitations to this investigation. In our previous study, which was based on admission data from the TTM-trial, ANN was superior to logistic regression in predicting long-term neurological outcome [20]. We chose the same approach in this study, well aware that the decreasing number of patients in the days following ICU admission may be too low for ANN models to perform well (see Fig. 1). With very strong biomarkers like NFL and a limited number of patients for model development, ANN might not be superior to logistic regression. Furthermore, the size of the dataset led to moderately wide confidence intervals in the test set and even though the training/test split was random, the size of the datasets made the test sets vulnerable to patient outliers and thereby affecting the model performance.

Moreover, differences in patient care during the last decade, from prehospital response to post-arrest care, could decrease the generalisability of the models, as our models are based on patient data from seven to ten years ago. There is, however, no new evidence-based therapies included in clinical practice during this period. Additionally, these models are based on a randomised trial which only included patients with OHCA of a presumed cardiac cause which could affect the generalisability.

Another limitation of this study is that none of the important prognostic examinations such as EEG, SSEP or neuroradiological imaging after admission were included. When only incorporating clinical variables (neither clinically accessible nor research-grade biomarkers), the prognostic performance did not improve in the first three days after ICU admission (see Fig. 2). When comparing the prognostic performance to prediction models based on merely prehospital and admission data, it seems little, if any, prognostic value is added after hospital admission using only clinical variables (approximately 20 variables in each model, see Table 1) [20, 36, 37]. The TTM risk score and the ANN model in our previous study were developed on the same population as this study and showed good prognostic performance with AUROC of 84.2% and 89.1%, respectively [20, 36]. It is a noteworthy finding that the prognostic improvement is absent during the first three days after ICU admission. This underlines the need to use other prognostic tools like SSEP, EEG etc. when performing cardiac arrest prognostication in an ICU setting. Furthermore, it also pinpoints the uncertainty when using datasets of the size. When evaluating the model performance on the independent test set, the results can be affected by the training/test split and be vulnerable to outliers. For example, the model performance was presumably better at the time of hospital admission than after 24 h without biomarkers [20]. This difference is important and must be kept in mind when discussing this approach to cardiac arrest prognostication.

From an ICU perspective, one of the strengths of this study was that we modified the study population and excluded patients who were either deceased or woke up during the first 72 h after ICU admission. This strategy distilled the dataset to those patients that were at risk of a poor prognosis at each time point. Without doing so, the prediction models would be falsely enhanced as we would be predicting patients who had already woken up.

To make these models ready for clinical implementation, external validation and the use of a larger population for model development is needed in order to minimise some of the limitations in this study.

## Conclusion

In this exploratory study, ANNs provided good to excellent prognostic accuracy in predicting neurological outcome in comatose patients post OHCA using clinical variables and biomarkers from the first three days of intensive care. The models which included NSE after 72 h and NFL on all days showed promising prognostic performance.

## Supplementary Information

**Additional file 1: Figure 4.** SHAP variable importance on day 1.

**Additional file 2: Figure 4.** SHAP variable importance on day 3.

**Additional file 3: Table 1A.** Baseline characteristics.

**Additional file 4: Table 1B.** Day 1–24 hours of ICU treatment.

**Additional file 5: Table 1C.** Day 2–48 hours of ICU treatment.

**Additional file 6: Table 1D.** Day 3–72 hours of ICU treatment.

## Data Availability

The data is available from the Target Temperature Management trial steering group after an approval process.

## References

[1] Cronberg T, Greer DM, Lilja G, Moulaert V, Swindell P, Rossetti AO (2020). Brain injury after cardiac arrest: from prognostication of comatose patients to rehabilitation. Lancet Neurol.

[2] Nolan JP, Soar J, Cariou A, Cronberg T, Moulaert VR, Deakin CD (2015). European resuscitation council and European society of intensive care medicine 2015 guidelines for post-resuscitation care. Intensive Care Med.

[3] Nolan JP, Cariou A (2015). Post-resuscitation care: ERC-ESICM guidelines 2015. Intensive Care Med.

[4] Sandroni C, D'Arrigo S, Nolan JP (2018). Prognostication after cardiac arrest. Crit Care.

[5] Moseby-Knappe M, Mattsson N, Nielsen N, Zetterberg H, Blennow K, Dankiewicz J (2019). Serum neurofilament light chain for prognosis of outcome after cardiac arrest. JAMA Neurol.

[6] Wihersaari L, Ashton NJ, Reinikainen M, Jakkula P, Pettilä V, Hästbacka J, et al. Neurofilament light as an outcome predictor after cardiac arrest: a post hoc analysis of the COMACARE trial. Intensive Care Medicine. 2020. 10.1007/s00134-020-06218-9 PMC7782453 32852582

[7] Kim MJ, Kim T, Suh GJ, Kwon WY, Kim KS, Jung YS (2018). Association between the simultaneous decrease in the levels of soluble vascular cell adhesion molecule-1 and S100 protein and good neurological outcomes in cardiac arrest survivors. Clin Exp Emerg Med.

[8] Mattsson N, Zetterberg H, Nielsen N, Blennow K, Dankiewicz J, Friberg H (2017). Serum tau and neurological outcome in cardiac arrest. Ann Neurol.

[9] Kaneko T, Kasaoka S, Miyauchi T, Fujita M, Oda Y, Tsuruta R (2009). Serum glial fibrillary acidic protein as a predictive biomarker of neurological outcome after cardiac arrest. Resuscitation.

[10] Ebner F, Moseby-Knappe M, Mattsson-Carlgren N, Lilja G, Dragancea I, Unden J, et al. Serum GFAP and UCH-L1 for the prediction of neurological outcome in comatose cardiac arrest patients. Resuscitation. 2020. 10.1016/j.resuscitation.2020.05.016 32445783

[11] Ok G, Aydin D, Erbuyun K, Gursoy C, Taneli F, Bilge S (2016). Neurological outcome after cardiac arrest: a prospective study of the predictive ability of prognostic biomarkers neuron-specific enolase, glial fibrillary acidic protein, S-100B, and procalcitonin. Turk J Med Sci.

[12] Annborn M, Nilsson F, Dankiewicz J, Rundgren M, Hertel S, Struck J (2016). The combination of biomarkers for prognostication of long-term outcome in patients treated with mild hypothermia after out-of-hospital cardiac arrest-a pilot study. Ther Hypothermia Temp Manag.

[13] Düring J, Annborn M, Cronberg T, Dankiewicz J, Devaux Y, Hassager C, et al. Copeptin as a marker of outcome after cardiac arrest: a sub-study of the TTM trial. Critical care (London, England). 2020;24(1):185-. 10.1186/s13054-020-02904-8 PMC7189642 32345356

[14] Myhre PL, Tiainen M, Pettila V, Vaahersalo J, Hagve TA, Kurola J (2016). NT-proBNP in patients with out-of-hospital cardiac arrest: Results from the FINNRESUSCI Study. Resuscitation.

[15] Bro-Jeppesen J, Kjaergaard J, Stammet P, Wise MP, Hovdenes J, Åneman A (2016). Predictive value of interleukin-6 in post-cardiac arrest patients treated with targeted temperature management at 33 °C or 36 °C. Resuscitation.

[16] Frydland M, Kjaergaard J, Erlinge D, Stammet P, Nielsen N, Wanscher M (2016). Usefulness of serum B-type natriuretic peptide levels in comatose patients resuscitated from out-of-hospital cardiac arrest to predict outcome. Am J Cardiol.

[17] Holmgren G, Andersson P, Jakobsson A, Frigyesi A (2019). Artificial neural networks improve and simplify intensive care mortality prognostication: a national cohort study of 217,289 first-time intensive care unit admissions. J Intensive Care.

[18] Majkowska A, Mittal S, Steiner DF, Reicher JJ, McKinney SM, Duggan GE (2020). Chest radiograph interpretation with deep learning models: assessment with radiologist-adjudicated reference standards and population-adjusted evaluation. Radiology.

[19] Kwon JM, Jeon KH, Kim HM, Kim MJ, Lim S, Kim KH (2019). Deep-learning-based out-of-hospital cardiac arrest prognostic system to predict clinical outcomes. Resuscitation.

[20] Johnsson J, Bjornsson O, Andersson P, Jakobsson A, Cronberg T, Lilja G (2020). Artificial neural networks improve early outcome prediction and risk classification in out-of-hospital cardiac arrest patients admitted to intensive care. Crit Care.

[21] Nielsen N, Wetterslev J, Cronberg T, Erlinge D, Gasche Y, Hassager C (2013). Targeted temperature management at 33 degrees C versus 36 degrees C after cardiac arrest. N Engl J Med.

[22] Nielsen N, Wetterslev J, Al-Subaie N, Andersson B, Bro-Jeppesen J, Bishop G (2012). Target temperature management after out-of-hospital cardiac arrest–a randomized, parallel-group, assessor-blinded clinical trial–rationale and design. Am Heart J..

[23] Nielsen N, Winkel P, Cronberg T, Erlinge D, Friberg H, Gasche Y (2013). Detailed statistical analysis plan for the target temperature management after out-of-hospital cardiac arrest trial. Trials.

[24] Cronberg T, Lilja G, Horn J, Kjaergaard J, Wise MP, Pellis T (2015). Neurologic function and health-related quality of life in patients following targeted temperature management at 33 degrees C vs 36 degrees C after out-of-hospital cardiac arrest: a randomized clinical trial. JAMA Neurol.

[25] A randomized clinical study of cardiopulmonary-cerebral resuscitation: design, methods, and patient characteristics. Brain Resuscitation Clinical Trial I Study Group. Am J Emerg Med. 1986;4(1):72–86. 2868736

[26] Keany E. BorutaShap 1.0.14 2020 [Available from: https://pypi.org/project/BorutaShap/.

[27] R Core Team. R: A language and environment for statistical computing. R Foundation for Statistical Computing; 2020.

[28] Python Core Team. Python: A dynamic, open source programming language. Python version 3.7 ed: Python Software Foundation; 2020.

[29] Abadi M, Barham P, Chen JM, Chen ZF, Davis A, Dean J, et al. TensorFlow: a system for large-scale machine learning. In: Proceedings of Osdi'16: 12th Usenix symposium on operating systems design and implementation. 2016:265–83.

[30] Lundberg SM, Lee S-I. A unified approach to interpreting model predictions. Proceedings of the 31st international conference on neural information processing systems; Long Beach, California, USA: Curran Associates Inc.; 2017. p. 4768–77.

[31] Robin X, Turck N, Hainard A, Tiberti N, Lisacek F, Sanchez JC (2011). pROC: an open-source package for R and S+ to analyze and compare ROC curves. BMC Bioinformatics.

[32] López-Ratón M, Rodríguez-Álvarez MX, Suárez CC, Sampedro FG (2014). OptimalCutpoints: an R package for selecting optimal cutpoints in diagnostic tests. J Stat Softw.

[33] Collins GS, Reitsma JB, Altman DG, Moons KG (2015). Transparent reporting of a multivariable prediction model for individual prognosis or diagnosis (TRIPOD): the TRIPOD statement. Ann Int Med.

[34] Moseby-Knappe M, Westhall E, Backman S, Mattsson-Carlgren N, Dragancea I, Lybeck A, et al. Performance of a guideline-recommended algorithm for prognostication of poor neurological outcome after cardiac arrest. Intensive Care Med. 2020. 10.1007/s00134-020-06080-9 PMC7527324 32494928

[35] Stammet P, Collignon O, Hassager C, Wise MP, Hovdenes J, Aneman A (2015). Neuron-specific enolase as a predictor of death or poor neurological outcome after out-of-hospital cardiac arrest and targeted temperature management at 33 degrees C and 36 degrees C. J Am Coll Cardiol.

[36] Martinell L, Nielsen N, Herlitz J, Karlsson T, Horn J, Wise MP, et al. Early predictors of poor outcome after out-of-hospital cardiac arrest. Critical care (London, England). 2017;21(1):96-. 10.1186/s13054-017-1677-2 PMC5391587 28410590

[37] Pareek N, Kordis P, Beckley-Hoelscher N, Pimenta D, Kocjancic ST, Jazbec A, et al. A practical risk score for early prediction of neurological outcome after out-of-hospital cardiac arrest: MIRACLE2. Eur Heart J. 2020. 10.1093/eurheartj/ehaa570 32731260

