# Supplementary figures and images for: Predicting neurological outcome after out-of-hospital cardiac arrest with cumulative information; development and internal validation of an artificial neural network algorithm
Source: Crit Care. 2021 Feb 25;25:83. doi: 10.1186/s13054-021-03505-9 (PMC7905905; doi:10.1186/s13054-021-03505-9)

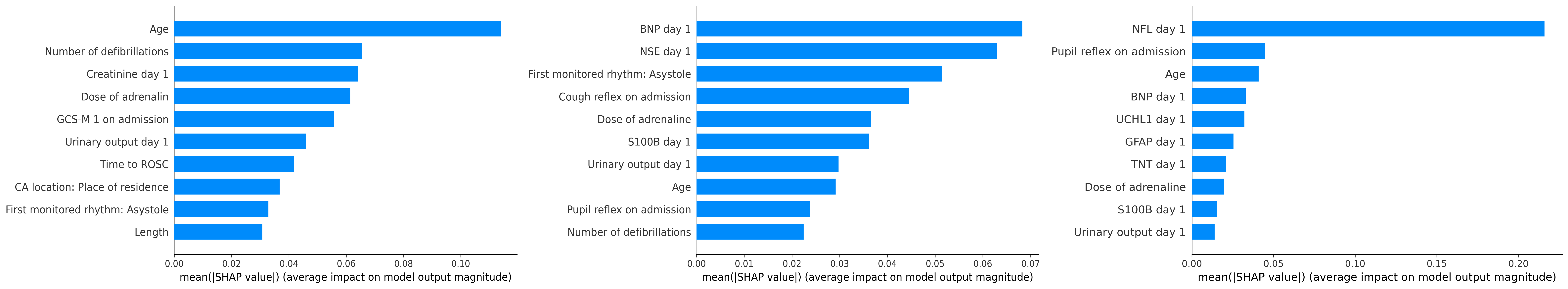

Supplement: Supplementary file 1 — Additional file 1: Figure 4. SHAP variable importance on day 1. [file 13054_2021_3505_MOESM1_ESM.png]

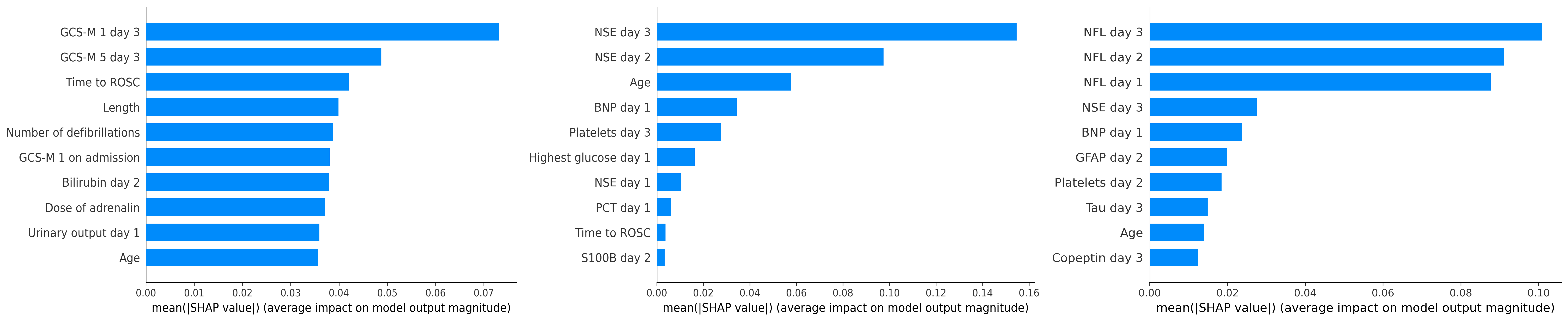

Supplement: Supplementary file 2 — Additional file 2: Figure 4. SHAP variable importance on day 3. [file 13054_2021_3505_MOESM2_ESM.png]
