# Supplementary material for: Predicting neurological outcome after out-of-hospital cardiac arrest with cumulative information; development and internal validation of an artificial neural network algorithm
Source: Crit Care. 2021 Feb 25;25:83. doi: 10.1186/s13054-021-03505-9 (PMC7905905; doi:10.1186/s13054-021-03505-9)
Supplement: Supplementary file 3 — Additional file 3: Table 1A. Baseline characteristics. [file 13054_2021_3505_MOESM3_ESM.docx]

**Table 1A. Baseline characteristics**

|  | **CPC score 1-2** | **CPC score 3-5** | ***p*-value** | **Missing (%)** |
| --- | --- | --- | --- | --- |
| **Background**  No. of patients  Age, years (IQR)  Female sex (%)  Length, cm (IQR)  Weight, kg (IQR)  Chronic heart failure (%)  Previous myocardial infarction (%)  Ischaemic heart disease (%)  Previous cardiac arrhythmia (%)  Previous cardiac arrest (%)  Arterial hypertension (%)  TIA or stroke (%)  Epilepsy (%)  Diabetes (%)  Asthma or COPD (%)  Chronic dialysis (%)  Cirrhosis (%)  Haematological malignancy (%)  AIDS  IV drug abuse  Immunodeficiency  Other malignancy (%)  Alcoholism (%)  Previous PCI (%)  Previous CABG (%)  Previous valvular surgery (%)  Implantable cardioverter-defibrillator (ICD) (%)  Pacemaker (%)  Pre-arrest CPC  CPC 1  CPC 2  CPC 3 | 440  61 (52-69)  66 (15.0)  179 (171-183)  80 (73-90)  16 (3.6)  79 (18.0)  101 (23.0)  60 (13.6)  9 (2.0)  150 (34.2)  23 (5.2)  11 (2.5)  51 (11.6)  31 (7.0)  2 (0.5)  0 (0.0)  2 (0.5)  1 (0.2)  2 (0.5)  2 (0.5)  7 (1.6)  10 (2.3)  45 (10.2)  26 (5.9)  10 (2.3)  1 (0.2)  11 (2.5)  430 (97.7)  10 (2.3)  0 (0.0) | 492  68 (61-76)  111 (22.6)  175 (167-180)  80 (70-90)  44 (9.0)  112 (22.8)  157 (32.0)  103 (21.0)  12 (2.4)  222 (45.3)  50 (10.2)  5 (1.0)  89 (18.2)  65 (13.2)  4 (0.8)  3 (0.6)  7 (1.4)  0 (0.0)  2 (0.4)  2 (0.4)  16 (3.3)  26 (5.3)  62 (12.7)  62 (12.7)  15 (3.1)  4 (0.8)  21 (4.3)  457 (94.0)  28 (5.8)  1 (0.2) | <0.001  0.004  <0.001  0.207  0.001  0.080  0.003  0.004  0.851  0.001  0.007  0.135  0.007  0.003  0.785  0.288  0.241  0.954  1.000  1.000  0.156  0.027  0.286  0.001  0.590  0.444  0.196  0.018 | 0.0  0.0  2.4  1.4  0.2  0.1  0.2  0.1  0.1  0.3  0.3  0.1  0.5  0.0  0.0  0.0  0.6  0.8  0.2  0.2  0.4  0.1  0.3  0.3  0.4  0.3  0.3  0.6 |
| **Prehospital**  Cardiac arrest location (%)  Place of residence  Public place  Other  Bystander witnessed arrest (%)  Bystander CPR (%)  Bystander defibrillation (%)  First monitored rhythm (%)  Non-perfusing ventricular tachycardia (VT)  Ventricular fibrillation (VF)  Asystole  Pulseless electrical activity (PEA)  Unknown  ROSC after bystander defibrillation  First rhythm shockable (%)  Automatic compression-decompression (%)  No  Yes, manual  Yes, mechanical  Number of defibrillations (IQR)  Pre-hospital intubation (%)  Seizures before admission (%)  No  Yes, before CA  Yes, after resuscitation  Total dose of adrenaline, mg (IQR)  CA to BLS, min (IQR) | 192 (43.6)  216 (49.1)  32 (7.3)  406 (92.3)  347 (78.9)  55 (12.5)  11 (2.5)  391 (88.9)  12 (2.7)  12 (2.7)  4 (0.9)  10 (2.3)  414 (94.1)  348 (79.1)  30 (6.8)  62 (14.1)  3 (1-4)  273 (62.9)  406 (92.5)  21 (4.8)  12 (2.7)  1 (0-3)  1 (0-2) | 306 (62.2)  166 (33.7)  20 (4.1)  427 (86.8)  331 (67.4)  34 (6.9)  12 (2.4)  311 (63.2)  100 (20.3)  53 (10.8)  14 (2.8)  2 (0.4)  333 (67.7)  363 (74.1)  35 (7.1)  92 (18.8)  2 (1-4)  352 (72.6)  468 (95.3)  6 (1.2)  17 (3.5)  3 (1-5)  1 (0-3) | <0.001  0.009  <0.001  0.005  <0.001  <0.001  0.145  0.001  0.002  0.005  <0.001  0.003 | 0.0  0.0  0.1  0.1  0.0  0.0  0.2  0.5  1.4  0.2  0.4  22.9 |
| CA to ALS, min (IQR)  CA to ROSC, min (IQR) | 8 (5-11)  20 (15-30) | 10 (7-15)  31 (21-47) | <0.001  <0.001 | 1.5  0.0 |
|  |  |  |  |  |
| **Admission**  Initial temperature, °C (IQR)  Thrombolysis on admission (%)  Shock on admission (%)^a^  Glasgow coma scale (GCS) motor score = 1 (%)  Pupil reflex, present (%)  Corneal reflex, present (%)  Spontaneous breathing, present (%)  Cough reflex, present (%)  ECG findings (%)  Acute LBBB  Acute ST-elevation  Atrial fibrillation-flutter  Normal or unchanged from previous ECGs  Other abnormality  Blood-glucose, mmol/L (IQR)  FiO_2_, % (IQR)  PaO_2_, kPa (IQR) ^b^  PaCO_2_, kPa (IQR) ^c^  pH (IQR)  Lactate, mmol/L (IQR)  Base excess, BE (IQR)  Potassium, mmol/L (IQR)  Creatinine, µmol/L (IQR)  Platelets, cells x 10^9^ (IQR)  WBC, cells x 10^9^ (IQR)  GCS - Eye-opening (%)  1  2  3  4  Sedation affecting GCS evaluation  GCS – Verbal (%)  1  2  3  4  5  Intubated  Sedation affecting GCS evaluation  GCS – Motor (%)  1  2  3  4  5  6  Sedation affecting GCS evaluation | 35.5 (34.9-36.0)  10 (2.3)  36 (8.2)  173 (39.4)  348 (88.3)  298 (78.2)  310 (72.9)  277 (70.1)  20 (4.6)  197 (45.0)  22 (5.0)  105 (24.0)  94 (21.5)  12.6 (9.5-16.0)  80 (50-100)  18.3 (11.7-30.1)  6.0 (5.2-6.8)  7.27 (7.17-7.32)  4.6 (2.4-8.1)  -6.0 (-10.0 - -4.0)  3.7 (3.4-4.2)  95 (80-115)  220 (185-265)  14.0 (10.6-18.0)  265 (60.4)  11 (2.5)  3 (0.7)  7 (1.6)  153 (34.9)  120 (27.4)  17 (3.9)  2 (0.5)  0 (0)  0 (0)  195 (44.5)  104 (23.7)  173 (39.4)  22 (0.5)  34 (7.7)  61 (13.9)  0 (0)  1 (0.2)  148 (33.7) | 35.3 (34.4-36.0)  9 (1.8)  100 (20.3)  316 (64.9)  315 (66.3)  219 (53.3)  284 (60.3)  211 (48.6)  35 (7.2)  185 (38.1)  39 (0.8)  112 (23.1)  114 (23.5)  14.0 (10.6-18.0)  90 (53-100)  18.9 (12.1-37.1)  6.3 (5.2-7.8)  7.19 (7.05-7.28)  6.9 (3.9-10.6)  -10.0 (-14.5 - -5.0)  4.0 (3.5-4.5)  115 (90-140)  215 (170-274)  14.0 (10.4-18.5)  357 (73.2)  3 (0.6)  0 (0)  3 (0.6)  125 (25.6)  147 (30.2)  3 (0.6)  0 (0)  0 (0)  0 (0)  253 (52.0)  84 (17.2)  316 (64.9)  17 (3.5)  11 (2.3)  23 (4.7)  0 (0)  0 (0)  120 (24.6) | 0.002  0.809  <0.001  <0.001  <0.001  <0.001  <0.001  <0.001  0.061  <0.001  0.215  0.344  0.003  <0.001  <0.001  <0.001  <0.001  <0.001  0.128  0.872  <0.001  <0.001  <0.001 | 3.6  0.3  0.0  0.6  2.4  15.0  3.9  11.1  1.0  5.5  3.2  7.4  5.8  4.6  6.3  7.0  2.9  3.1  3.1  4.1  0.5  0.8  0.6 |

**Table 1A. Baseline characteristics for good outcome (CPC 1-2) and poor outcome (CPC3-5) patients after six months**

Data are presented as *n* (%) or median (IQR). *n* denotes the number of cases with valid data. A *p*-value of <0.05 was considered significant. The variables are grouped into background-, pre-hospital and admission variables. IQR, Interquartile range. AIDS, Acquired Immunodeficiency syndrome. CPC, Cerebral performance category. TIA, Transient ischemic attack. COPD, Chronic obstructive pulmonary disease. PCI, Percutaneous coronary intervention. CABG, Coronary artery bypass grafting. VT, Ventricular tachycardia. VF, Ventricular fibrillation. PEA, Pulseless electric activity. ROSC, Return of spontaneous circulation. CA, Cardiac arrest, BLS, Basic life support. ALS, Advanced life support. CPR, Cardiopulmonary Resuscitation. LBBB, Left bundle branch block. WBC, White blood cell. GCS, Glasgow coma scale.

^a^ Shock on admission was defined as systolic blood pressure of less than 90 mmHg for more than 30 min or end-organ hypoperfusion unless vasoactive drugs were administered. ^b^ PaO_2_ >60 kPa was changed to 60 kPa. ^c^ PaCO_2_ >20 kPa was changed to 20 kPa.
