# Supplementary material for: Predicting neurological outcome after out-of-hospital cardiac arrest with cumulative information; development and internal validation of an artificial neural network algorithm
Source: Crit Care. 2021 Feb 25;25:83. doi: 10.1186/s13054-021-03505-9 (PMC7905905; doi:10.1186/s13054-021-03505-9)
Supplement: Supplementary file 4 — Additional file 4: Table 1B. Day 1–24 hours of ICU treatment. [file 13054_2021_3505_MOESM4_ESM.docx]

**Table 1B. Day 1 – 24 hours of ICU treatment**

|  | **CPC score 1-2**  **(n=440)** | **CPC score 3-5**  **(n=492)** | ***p*-value** | **Missing (%)** |
| --- | --- | --- | --- | --- |
| **Standard ICU observation variables (level A)**  Lowest PaO_2,_ kPa (IQR)^a^  Highest FiO_2_, % (IQR)^a^  Bilirubin, µmol/L (IQR)  Platelets, 10^9^/L (IQR)  Creatinine, µmol/L (IQR)  CRP, mg/L (IQR)  INR (IQR) ^b^  Lactate, mmol/L (IQR)  Hemodynamic mechanical support (%)  Intra-aortic balloon pump (IABP)  No mechanical assist  Other device  Mechanical ventilation (%)  Renal replacement therapy (%)  Urinary output, ml (IQR)  Blood units, n (IQR)  Plasma units, n (IQR)  Fluid balance, ml (IQR)  Echocardiography (%)  EF normal or preserved (>50%)  EF moderately impaired (30-50%)  EF severely impaired (<30%)  Not performed  Cardiovascular function (MAP>70 mmHg) (%)  Inotropes used (%)  - Any dose of Dobutamine or <5microg/kg/min of Dopamine  - Dopamine 5-15 microg/kg/min or Noradrenaline/adrenaline <0.1 g/kg/min  - Dopamine >15 microg/kg/min or Noradrenaline/adrenaline >0.1 microg/kg/min  - Noradrenalin/adrenaline >0.25 microg/kg/min  - Noradrenalin/adrenaline >0.5 microg/kg/min  - Noradrenalin/adrenaline >0.75 microg/kg/min  - Noradrenalin/adrenaline >1.0 microg/kg/min  - No inotropic drug or vasopressor  Myoclonic seizures treatment (increased sedation) (%)  Tonic-clonic seizures treatment (increased sedation) (%)  Uncontrolled bleeding (%)  Intracerebral bleeding (%)  Intraspinal bleeding (%)  Intraocular bleeding (%)  Intraarticular bleed (%)  Pericardial bleeding (%)  Gastrointestinal bleeding (%)  Tracheal bleeding (%)  Oral bleeding (%)  Nose bleeding (%)  Genital bleeding (%)  Insertion bleeding (%)  Pneumonia (%)  No  Yes, confirmed  Yes, suspected  Severe sepsis (%)  No  Yes, confirmed  Yes, suspected  Septic shock (%)  No  Yes, confirmed  Yes, suspected  Other infection (%)  No  Yes, confirmed  Yes, suspected  Atrial fibrillation (%)  Atrial flutter (%)  Tachycardia (%)  Bradycardia (%)  VT (%)  VF (%)  CPR performed (%)  Lowest potassium, mmol/L (IQR)  Lowest magnesium, mmol/L (IQR)  Lowest phosphate, mmol/L (IQR)  Lowest glucose, mmol/L (IQR)  Highest glucose, mmol/L (IQR)  Shivering (%)  Highest body temperature, °C (IQR)  Time over 38°C, hours (IQR)  GCS - Eye-opening (%)  1  2  3  4  Sedation affecting GCS evaluation  GCS – Verbal (%)  1  2  3  4  5  Intubated  Sedation affecting GCS evaluation  GCS – Motor (%)  1  2  3  4  5  6  Sedation affecting GCS evaluation | 11.6 (9.8-14.7)  50 (35-60)  10 (7, 15)  200 (165-244)  95 (75-115)  5.0 (2.0-13.0)  1.20 (1.10-1.30)  2.75 (1.70-4.90)  60 (13.9)  362 (84.0)  9 (2.1)  435 (98.9)  7 (1.6)  1350 (750-2400)  0 (0-0)  0 (0-0)  1300 (400-2550)  65 (14.9)  133 (30.5)  67 (15.4)  171 (39.2)  184 (43.1)  53 (12.2)  149 (34.4)  94 (21.7)  17 (3.9)  11 (2.5)  1 (0.2)  3 (0.7)  105 (24.2)  4 (80.0)  1 (50.0)  5 (1.1)  1 (0.2)  0 (0.0)  0 (0.0)  0 (0.0)  2 (0.5)  12 (2.8)  8 (1.8)  13 (3.0)  12 (2.8)  2 (0.5)  13 (3.0)  394 (89.5)  10 (2.3)  36 (8.2)  437 (99.3)  2 (0.5)  1 (0.2)  437 (99.5)  2 (0.5)  0 (0.0)  436 (99.8)  0 (0.0)  1 (0.2)  49 (11.1)  5 (1.1)  14 (3.2)  16 (3.7)  46 (10.5)  13 (3.0)  14 (3.2)  3.50 (3.20-3.80)  0.80 (0.70-0.90)  1.10 (0.80-1.50)  6.5 (5.6-7.7)  10.7 (8.5-14.0)  82 (18.6)  36.1 (35.3-36.5)  0.0 (0.0-0.0)  47 (10.7)  0 (0.0)  2 (0.5)  4 (0.9)  386 (87.9)  12 (2.7)  1 (0.2)  0 (0)  0 (0)  1 (0.2)  154 (35.2)  269 (61.6)  36 (8.2)  4 (0.9)  6 (1.4)  3 (0.7)  2 (0.5)  4 (0.9)  383 (87.4) | 11.3 (9.75-14.6)  50 (40-70)  9 (6, 14)  200 (160-255)  115 (90-145)  7.0 (2.0-23.0)  1.20 (1.10-1.60)  4.90 (2.60-8.00)  65 (13.3)  416 (85.1)  8 (1.6)  485 (98.8)  23 (4.7)  900 (400-1165)  0 (0-0)  0 (0-0)  1700 (500-2975)  67 (14.0)  122 (25.5)  81 (16.9)  209 (43.6)  188 (38.7)  51 (10.5)  140 (28.7)  92 (18.9)  61 (12.5)  22 (4.5)  6 (1.2)  16 (3.3)  100 (20.5)  47 (71.2)  8 (61.5)  3 (0.6)  3 (0.6)  0 (0.0)  1 (0.2)  0 (0.0)  2 (0.4)  13 (2.7)  7 (1.5)  21 (4.3)  14 (2.9)  5 (1.0)  20 (4.1)  433 (88.2)  4 (0.8)  54 (11.0)  480 (98)  1 (0.2)  9 (1.8)  481 (98.0)  2 (0.4)  8 (1.6)  487 (99.2)  2 (0.4)  2 (0.4)  86 (17.6)  8 (1.6)  23 (4.7)  21 (4.3)  40 (8.2)  25 (5.1)  38 (7.8)  3.50 (3.20-3.90)  0.80 (0.70-1.00)  1.40 (0.90-2.10)  7.2 (5.8-9.1)  12.45 (9.7-16.4)  69 (14.1)  36.0 (34.8-36.5)  0.0 (0.0-0.0)  81 (16.5)  1 (0.2)  2 (0.4)  0 (0.0)  406 (82.9)  33 (6.7)  0 (0.0)  0 (0)  0 (0)  0 (0.0)  167 (34.1)  290 (59.2)  74 (15.1)  5 (1.0)  6 (1.2)  4 (0.8)  0 (0.0)  1 (0.2)  400 (81.6) | 0.295  0.001  0.014  0.917  <0.001  0.001  <0.001  <0.001  0.839  1.000  0.013  <0.001  0.011  0.429  0.017  0.306  0.199  <0.001  1.000  1.000  0.617  0.693  NA  1.000  NA  1.000  1.000  0.838  0.368  1.000  0.541  0.452  0.073  0.048  0.027  0.365  0.007  0.711  0.307  0.722  0.278  0.136  0.004  0.102  <0.001  <0.001  <0.001  <0.001  0.078  0.025  0.940  0.019  0.038  0.023 | 1.1  1.4  10.8  2.6  1.9  13.8  6.1  2.6  1.3  0.1  0.2  1.9  1.0  1.1  2.5  1.8  2.0  1.2  92.4  98.4  0.8  1.6  1.6  1.6  1.9  2.0  1.7  1.7  1.7  1.6  2.3  1.6  0.1  0.2  0.2  0.4  0.4  0.9  0.9  1.0  1.0  0.8  0.9  1.4  16.2  14.2  1.6  1.3  0.4  1.2  1.4  0.3  0.5  0.4 |
| **Clinically accessible biomarkers (level B)**  BNP, ng/L (IQR)  NSE, ng/ml (IQR)  PCT, µg/L (IQR)  S100B, µg/L (IQR)  TNT, ng/L (IQR) | 1092 (547-2101)  18.0 (12.4-26.6)  0.53 (0.21-1.91)  0.08 (0.06-0.11)  0.74 (0.16-2.41) | 2407 (1245-5182)  34.7 (20.6-58.8)  2.94 (0.73-9.50)  0.19 (0.10-0.50)  0.81 (0.20-2.99) | <0.001  <0.001  <0.001  <0.001  0.225 | 31.0  31.0  29.5  30.4  31.1 |
| **Research-grade biomarkers (level C)**  Copeptin, pmol/L (IQR)  IL6, ng/L (IQR)  NFL, ng/L (IQR)  Tau, ng/L (IQR)  GFAP, ng/L (IQR)  UCHL1, ng/L (IQR) | 15.3 (9.3-30.6)  168 (84-335)  37 (20-70)  2.4 (1.5-5.3)  27 (15-47)  240 (148-390) | 50.3 (19.6-96.6)  175 (86-358)  1421 (300-3573)  11.9 (4.3-44.2)  111 (60-380)  1130 (465-2780) | <0.001  0.277  <0.001  <0.001  <0.001  <0.001 | 29.6  30.9  25.4  25.2  25.1  25.1 |

**Table 1B. Variables collected during the first 24 hours of intensive care observation and treatment for good outcome (CPC 1-2) and poor outcome (CPC 3-5) patients after six months**

The variables are grouped into standard ICU observation variables, clinically accessible biomarkers and research-grade biomarkers, all collected during the TTM-trial. Data are presented as *n* (%) or median (IQR). *n* denotes the number of cases with valid data. A *p*-value of <0.05 was considered significant. ICU, Intensive care unit. CPC, Cerebral performance category. IQR, Interquartile range. CRP, C-reactive protein (mg/L). INR, International normalized ratio. EF, Ejection fraction. MAP, Mean arterial pressure. VT, Ventricular tachycardia. VF, Ventricular fibrillation. CPR, Cardiopulmonary Resuscitation. GCS, Glasgow coma scale. BNP, Brain natriuretic peptide (ng/L). NSE, Neuron-specific enolase (ng/ml). PCT, Procalcitonin. S100B, S100 calcium-binding protein B. TNT, Troponin T. IL6, Interleukin 6. NFL, Neurofilament light. GFAP, Glial fibrillary acidic protein. UCHL1, Ubiquitin carboxy-terminal hydrolase L1.

^a^Lowest PaO2 (kPa) with the corresponding highest FiO2 (%), PaO_2_ >60 kPa was changed to 60 kPa. ^b^ INR >10 was changed to 10.
