# Supplementary material for: Predicting neurological outcome after out-of-hospital cardiac arrest with cumulative information; development and internal validation of an artificial neural network algorithm
Source: Crit Care. 2021 Feb 25;25:83. doi: 10.1186/s13054-021-03505-9 (PMC7905905; doi:10.1186/s13054-021-03505-9)
Supplement: Supplementary file 5 — Additional file 5: Table 1C. Day 2–48 hours of ICU treatment. [file 13054_2021_3505_MOESM5_ESM.docx]

**Table 1C. Day 2 – 48 hours of ICU treatment**

|  | **CPC score 1-2**  **(n=440)** | **CPC score 3-5**  **(n=492)** | ***p*-value** | **Missing (%)** |
| --- | --- | --- | --- | --- |
| **Standard ICU observation variables (level A)**  Lowest PaO_2,_ kPa (IQR)^a^  Highest FiO_2_, % (IQR)^a^  Bilirubin, µmol/L (IQR)  Platelets, 10^9^/L (IQR)  Creatinine, µmol/L (IQR)  CRP, mg/L (IQR)  INR (IQR) ^b^  Lactate, mmol/L (IQR)  Hemodynamic mechanical support (%)  Intra-aortic balloon pump (IABP)  No mechanical assist  Other device  Mechanical ventilation(%)  Renal replacement therapy (%)  Urinary output, ml (IQR)  Blood units, n (IQR)  Plasma units, n (IQR)  Fluid balance, ml (IQR)  Echocardiography (%)  EF normal or preserved (>50%)  EF moderately impaired (30-50%)  EF severely impaired (<30%)  Not performed  Cardiovascular function (MAP>70 mmHg) (%)  Inotropes used (%)  - Any dose of Dobutamine or <5microg/kg/min of Dopamine  - Dopamine 5-15 microg/kg/min or Noradrenaline/adrenaline <0.1 g/kg/min  - Dopamine >15 microg/kg/min or Noradrenaline/adrenaline >0.1 microg/kg/min  - Noradrenalin/adrenaline >0.25 microg/kg/min  - Noradrenalin/adrenaline >0.5 microg/kg/min  - Noradrenalin/adrenaline >0.75 microg/kg/min  - Noradrenalin/adrenaline >1.0 microg/kg/min  - No inotropic drug or vasopressor  Myoclonic seizures treatment (increased sedation) (%)  Tonic-clonic seizures treatment (increased sedation) (%)  Uncontrolled bleeding (%)  Intracerebral bleeding (%)  Intraspinal bleeding (%)  Intraocular bleeding (%)  Intraarticular bleed (%)  Pericardial bleeding (%)  Gastrointestinal bleeding (%)  Tracheal bleeding (%)  Oral bleeding (%)  Nose bleeding (%)  Genital bleeding (%)  Insertion bleeding (%)  Pneumonia (%)  No  Yes, confirmed  Yes, suspected  Severe sepsis (%)  No  Yes, confirmed  Yes, suspected  Septic shock (%)  No  Yes, confirmed  Yes, suspected  Other infection (%)  No  Yes, confirmed  Yes, suspected  Atrial fibrillation (%)  Atrial flutter (%)  Tachycardia (%)  Bradycardia (%)  VT (%)  VF (%)  CPR performed (%)  Lowest potassium, mmol/L (IQR)  Lowest magnesium, mmol/L (IQR)  Lowest phosphate, mmol/L (IQR)  Lowest glucose, mmol/L (IQR)  Highest glucose, mmol/L (IQR)  Shivering (%)  Highest body temperature, °C (IQR)  Time over 38°C, hours (IQR)  GCS - Eye-opening (%)  1  2  3  4  Sedation affecting GCS evaluation  GCS – Verbal (%)  1  2  3  4  5  Intubated  Sedation affecting GCS evaluation  GCS – Motor (%)  1  2  3  4  5  6  Sedation affecting GCS evaluation | 10.2 (9.1-11.8)  35 (30-45)  12 (9, 17)  165 (135-200)  75 (60-100)  64 (30-110)  1.10 (1.10-1.30)  2.00 (1.30-3.00)  53 (12.3)  369 (85.6)  9 (2.1)  428 (97.7)  14 (3.2)  2200 (1500-3200)  0 (0-0)  0 (0-0)  1450 (500-2300)  35 (8.1)  92 (21.2)  31 (7.2)  275 (63.5)  172 (40.1)  59 (13.7)  165 (38.2)  94 (21.8)  27 (6.2)  10 (2.3)  4 (0.9)  3 (0.7)  70 (16.2)  4 (80.0)  1 (50.0)  2 (0.5)  1 (0.2)  0 (0.0)  0 (0.0)  0 (0.0)  2 (0.5)  13 (3.0)  6 (1.4)  17 (3.9)  10 (2.3)  3 (0.7)  17 (3.9)  333 (75.9)  23 (5.2)  83 (18.9)  423 (96.6)  7 (1.6)  8 (1.8)  432 (98.6)  2 (0.5)  4 (0.9)  436 (99.8)  0 (0.0)  1 (0.2)  29 (6.6)  3 (0.7)  10 (2.3)  14 (3.2)  30 (6.8)  8 (1.8)  6 (1.4)  3.60 (3.40-3.90)  0.80 (0.70-0.90)  0.90 (0.70-1.10)  5.7 (5.0-6.4)  8.8 (7.5-10.5)  87 (19.9)  37.0 (36.2-37.5)  0.0 (0.0-0.0)  34 (7.8)  2 (0.5)  6 (1.4)  40 (9.1)  356 (81.3)  13 (3.0)  1 (0.2)  1 (0.2)  14 (3.2)  9 (2.1)  164 (37.4)  237 (54.0)  28 (6.4)  1 (0.2)  2 (0.5)  12 (2.7)  4 (0.9)  33 (7.5)  359 (81.8) | 10.1 (9.0-11.6)  40 (30-50)  10 (7, 15)  175 (135-225)  105 (75-155)  72 (32-117)  1.20 (1.10-1.40)  2.50 (1.70-4.60)  57 (12.5)  393 (86.2)  6 (1.3)  450 (98.5)  39 (8.6)  1700 (900-2600)  0 (0-0)  0 (0-0)  1700 (600-2800)  32 (7.2)  67 (15.2)  41 (9.3)  302 (68.3)  168 (37.4)  34 (7.5)  143 (31.4)  92 (20.2)  69 (15.2)  21 (4.6)  9 (2.0)  16 (3.5)  71 (15.6)  49 (55.7)  14 (73.7)  3 (0.7)  3 (0.7)  1 (0.2)  1 (0.2)  1 (0.2)  2 (0.5)  11 (2.5)  5 (1.1)  17 (3.8)  10 (2.2)  4 (0.9)  18 (4.0)  363 (79.8)  15 (3.3)  77 (16.9)  433 (95.2)  2 (0.4)  20 (4.4)  436 (95.8)  3 (0.7)  16 (3.5)  452 (98.9)  2 (0.4)  3 (0.7)  69 (15.1)  5 (1.1)  21 (4.6)  13 (2.9)  34 (7.5)  10 (2.2)  11 (2.4)  3.70 (3.40-4.00)  0.90 (0.70-1.00)  1.00 (0.70-1.30)  5.8 (4.9-6.8)  9.4 (7.9-11.9)  67 (14.7)  37.0 (35.9-37.5)  0.0 (0.0-0.0)  68 (14.9)  3 (0.7)  5 (1.1)  16 (3.5)  365 (79.9)  32 (7.0)  0 (0.0)  0 (0.0)  1 (0.2)  1 (0.2)  154 (33.8)  268 (58.8)  75 (16.4)  3 (0.7)  1 (0.2)  6 (1.3)  2 (0.4)  6 (1.3)  363 (79.6) | 0.364  0.003  <0.001  0.058  <0.001  0.120  <0.001  <0.001  0.671  0.563  0.001  <0.001  0.031  0.925  0.023  0.085  0.457  <0.001  0.546  1.000  1.000  0.637  1.000  1.000  1.000  1.000  0.783  0.958  1.000  1.000  1.000  1.000  0.233  0.021  0.029  0.241  <0.001  0.763  0.086  0.925  0.810  0.872  0.368  0.045  <0.001  <0.001  0.306  <0.001  0.050  0.055  0.181  <0.001  <0.001  <0.001 | 4.2  4.4  13.5  7.1  6.0  16.1  11.1  4.7  4.8  4.0  4.2  4.6  4.5  4.6  5.0  6.1  5.8  4.8  90.0  97.7  4.2  5.9  5.8  6.0  6.0  6.4  6.0  5.8  5.9  5.9  6.1  6.0  4.1  4.2  4.2  4.1  4.0  4.0  4.1  4.1  4.1  4.1  4.6  4.2  14.8  13.5  4.4  4.4  4.1  4.2  4.3  4.0  4.0  4.0 |
| **Clinically accessible biomarkers (level B)**  BNP, ng/L (IQR)  NSE, ng/ml (IQR)  PCT, µg/L (IQR)  S100B, µg/L (IQR)  TNT, ng/L (IQR) | 1177 (561-2500)  15 (10-21)  0.50 (0.21-1.53)  0.07 (0.06-0.11)  0.50 (0.12-1.89) | 2569 (1177-5174)  60 (24-125)  2.11 (0.61-6.86)  0.16 (0.10-0.43)  0.69 (0.18-2.19) | <0.001  <0.001  <0.001  <0.001  0.076 | 33.9  34.3  31.9  33.7  33.9 |
| **Research-grade biomarkers (level C)**  Copeptin, pmol/L (IQR)  IL6, ng/L (IQR)  NFL, ng/L (IQR)  Tau, ng/L (IQR)  GFAP, ng/L (IQR)  UCHL1, ng/L (IQR) | 29.0 (13.1-67.4)  131 (69-298)  46 (26-100)  2 (1-3)  29 (17-49)  204 (140-329) | 55.6 (24.9-93.2)  166 (90-445)  3257 (623-8265)  46 (7-337)  140 (71-480)  1178 (395-2909) | <0.001  0.001  <0.001  <0.001  <0.001  <0.001 | 32.1  32.7  29.1  28.9  28.4  28.3 |
|  |  |  |  |  |

**Table 1C. Variables collected between 24-48 hours of intensive care observation and treatment for good outcome (CPC 1-2) and poor outcome (CPC 3-5) patients after six months**

The variables are grouped into standard ICU observation variables, clinically accessible biomarkers and research-grade biomarkers, all collected during the TTM-trial. Data are presented as *n* (%) or median (IQR). *n* denotes the number of cases with valid data. A *p*-value of <0.05 was considered significant. ICU, Intensive care unit. CPC, Cerebral performance category. IQR, Interquartile range. CRP, C-reactive protein (mg/L). INR, International normalized ratio. EF, Ejection fraction. MAP, Mean arterial pressure. VT, Ventricular tachycardia. VF, Ventricular fibrillation. CPR, Cardiopulmonary Resuscitation. GCS, Glasgow coma scale. BNP, Brain natriuretic peptide. NSE, Neuron-specific enolase. PCT, Procalcitonin. S100B, S100 calcium-binding protein B. TNT, Troponin T. IL6, Interleukin 6. NFL, Neurofilament light. GFAP, Glial fibrillary acidic protein. UCHL1, Ubiquitin carboxy-terminal hydrolase L1.

^a^Lowest PaO2 (kPa) with the corresponding highest FiO2 (%), PaO_2_ >60 kPa was changed to 60 kPa. ^b^ INR >10 was changed to 10.
