# Supplementary material for: Predicting neurological outcome after out-of-hospital cardiac arrest with cumulative information; development and internal validation of an artificial neural network algorithm
Source: Crit Care. 2021 Feb 25;25:83. doi: 10.1186/s13054-021-03505-9 (PMC7905905; doi:10.1186/s13054-021-03505-9)
Supplement: Supplementary file 6 — Additional file 6: Table 1D. Day 3–72 hours of ICU treatment. [file 13054_2021_3505_MOESM6_ESM.docx]

**Table 1D. Day 3 – 72 hours of ICU treatment**

|  | **CPC score 1-2**  **(n=440)** | **CPC score 3-5**  **(n=492)** | ***p*-value** | **Missing (%)** |
| --- | --- | --- | --- | --- |
| **Standard ICU observation variables (level A)**  Lowest PaO_2,_ kPa (IQR)^a^  Highest FiO_2_, % (IQR)^a^  Bilirubin, µmol/L (IQR)  Platelets, 10^9^/L (IQR)  Creatinine, µmol/L (IQR)  CRP, mg/L (IQR)  INR (IQR) ^b^  Lactate, mmol/L (IQR)  Hemodynamic mechanical support (%)  Intra-aortic balloon pump (IABP)  No mechanical assist  Other devices  Mechanical ventilation (%)  Renal replacement therapy (%)  Urinary output, ml (IQR)  Blood units, n (IQR)  Plasma units, n (IQR)  Fluid balance, ml (IQR)  Echocardiography (%)  EF normal or preserved (>50%)  EF moderately impaired (30-50%)  EF severely impaired (<30%)  Not performed  Cardiovascular function (MAP>70 mmHg) (%)  Inotropes used (%)  - Any dose of Dobutamine or <5microg/kg/min of Dopamine  - Dopamine 5-15 microg/kg/min or Noradrenaline/adrenaline <0.1 g/kg/min  - Dopamine >15 microg/kg/min or Noradrenaline/adrenaline >0.1 microg/kg/min  - Noradrenalin/adrenaline >0.25 microg/kg/min  - Noradrenalin/adrenaline >0.5 microg/kg/min  - Noradrenalin/adrenaline >0.75 microg/kg/min  - Noradrenalin/adrenaline >1.0 microg/kg/min  - No inotropic drug or vasopressor  Myoclonic seizures treatment (increased sedation) (%)  Tonic-clonic seizures treatment (increased sedation) (%)  Uncontrolled bleeding (%)  Intracerebral bleeding (%)  Intraspinal bleeding (%)  Intraocular bleeding (%)  Intraarticular bleed (%)  Pericardial bleeding (%)  Gastrointestinal bleeding (%)  Tracheal bleeding (%)  Oral bleeding (%)  Nose bleeding (%)  Genital bleeding (%)  Insertion bleeding (%)  Pneumonia (%)  No  Yes, confirmed  Yes, suspected  Severe sepsis (%)  No  Yes, confirmed  Yes, suspected  Septic shock (%)  No  Yes, confirmed  Yes, suspected  Other infection (%)  No  Yes, confirmed  Yes, suspected  Atrial fibrillation (%)  Atrial flutter (%)  Tachycardia (%)  Bradycardia (%)  VT (%)  VF (%)  CPR performed (%)  Lowest potassium, mmol/L (IQR)  Lowest magnesium, mmol/L (IQR)  Lowest phosphate, mmol/L (IQR)  Lowest glucose, mmol/L (IQR)  Highest glucose, mmol/L (IQR)  Shivering (%)  Highest body temperature, °C (IQR)  Time over 38°C, hours (IQR)  GCS - Eye-opening (%)  1  2  3  4  Sedation affecting GCS evaluation  GCS – Verbal (%)  1  2  3  4  5  Intubated  Sedation affecting GCS evaluation  GCS – Motor (%)  1  2  3  4  5  6  Sedation affecting GCS evaluation | 9.7 (8.5-11.0)  40 (30-46)  11 (8, 16)  150 (120-185)  80 (65-104)  140 (96-196)  1.20 (1.10-1.30)  1.60 (1.20-2.30)  49 (11.4)  373 (86.7)  8 (1.9)  349 (80.0)  13 (3.0)  2700 (1750-3600)  0 (0-0)  0 (0-0)  100 (-700-1175)  23 (5.3)  65 (15.1)  20 (4.7)  322 (74.9)  228 (53.1)  53 (12.4)  126 (29.4)  71 (16.6)  25 (5.8)  5 (1.2)  3 (0.7)  5 (1.2)  141 (32.9)  7 (77.8)  3 (42.9)  3 (0.7)  1 (0.2)  0 (0.0)  0 (0.0)  0 (0.0)  1 (0.2)  5 (1.2)  6 (1.4)  10 (2.3)  7 (1.6)  2 (0.5)  14 (3.3)  272 (62.2)  57 (13.0)  108 (24.7)  413 (94.5)  12 (2.7)  12 (2.7)  428 (98.4)  1 (0.2)  6 (1.4)  432 (99.3)  0 (0.0)  3 (0.7)  40 (9.1)  2 (0.5)  18 (4.1)  10 (2.3)  17 (3.9)  3 (0.7)  3 (0.7)  3.70 (3.50-4.00)  0.80 (0.70-0.90)  1.00 (0.80-1.20)  5.8 (5.1-6.5)  8.4 (7.4-10.2)  50 (11.5)  37.8 (37.4-38.1)  0.0 (0.0-2.0)  27 (6.2)  13 (3.0)  51 (11.6)  205 (46.8)  142 (32.4)  14 (3.2)  11 (2.5)  19 (4.3)  69 (15.8)  46 (10.5)  180 (41.2)  98 (22.4)  21 (4.8)  1 (0.2)  4 (0.9)  35 (8.0)  46 (10.5)  189 (43.2)  142 (32.4) | 9.9 (8.8-11.0)  40 (30-50)  10 (7, 16)  155 (115-195)  115 (80-170)  147 (100-210)  1.20 (1.10-1.40)  2.10 (1.40-3.00)  47 (11.1)  375 (88.7)  1 (0.2)  405 (95.3)  41 (9.7)  1900 (1000-3000)  0 (0-0)  0 (0-0)  700 (-100-1800)  17 (4.0)  35 (8.3)  23 (5.5)  346 (82.2)  200 (47.5)  42 (9.9)  113 (26.7)  79 (18.6)  60 (14.2)  23 (5.4)  6 (1.4)  6 (1.4)  95 (22.4)  65 (51.6)  17 (70.8)  1 (0.2)  3 (0.7)  0 (0.0)  1 (0.2)  0 (0.0)  0 (0.0)  7 (1.7)  3 (0.7)  10 (2.4)  10 (2.4)  5 (1.2)  6 (1.4)  278 (65.4)  40 (9.4)  107 (25.2)  390 (92.4)  9 (2.1)  23 (5.5)  402 (95.0)  7 (1.7)  14 (3.3)  415 (97.9)  3 (0.7)  6 (1.4)  84 (19.9)  5 (1.2)  29 (6.9)  16 (3.8)  18 (4.3)  8 (1.9)  13 (3.1)  3.90 (3.70-4.30)  0.90 (0.80-1.00)  1.10 (0.90-1.40)  6.0 (5.0-6.9)  9.4 (7.9-10.9)  53 (12.5)  37.7 (37.3-38.1)  0.0 (0.0-1.5)  150 (35.3)  23 (5.4)  21 (4.9)  39 (9.2)  192 (45.2)  52 (12.2)  1 (0.2)  2 (0.5)  3 (0.7)  8 (1.9)  222 (52.2)  137 (32.2)  153 (36.0)  18 (4.2)  11 (2.6)  19 (4.5)  11 (2.6)  22 (5.2)  191 (44.9) | 0.403  0.025  0.061  0.207  <0.001  0.055  0.001  <0.001  0.066  <0.001  <0.001  <0.001  0.417  0.954  <0.001  0.012  0.115  <0.001  0.240  0.362  0.638  0.595  NA  0.988  NA  1.000  0.730  0.533  1.000  0.576  0.424  0.129  0.237  0.117  0.015  0.122  <0.001  0.421  0.105  0.280  0.911  0.202  0.020  <0.001  <0.001  <0.001  0.288  <0.001  0.728  0.065  0.903  <0.001  <0.001  <0.001 | 8.6  7.7  19.1  12.0  10.4  19.7  15.0  10.5  8.5  7.6  7.7  8.8  8.3  8.2  10.0  8.7  8.8  8.5  85.5  96.7  7.8  9.5  9.8  9.5  9.8  10.2  9.5  9.5  9.7  9.7  9.5  9.9  7.5  7.8  7.9  7.8  7.6  7.6  7.6  7.7  7.7  7.9  7.8  8.3  20.7  18.1  8.3  8.0  7.6  7.6  7.8  7.4  7.5  7.4 |
| **Clinically accessible biomarkers (level B)**  BNP, ng/L (IQR)  NSE, ng/ml (IQR)  PCT, µg/L (IQR)  S100B, µg/L (IQR)  TNT, ng/L (IQR) | 1926 (828-3411)  12 (9-17)  0.41 (0.17-1.35)  0.06 (0.04-0.09)  0.51 (0.10-1.85) | 2814 (1389-7127)  54 (19-133)  1.28 (0.43-3.94)  0.13 (0.08-0.26)  0.60 (0.14-1.72) | <0.001  <0.001  <0.001  <0.001  0.291 | 38.5  39.1  37.8  37.9  38.6 |
| **Research-grade biomarkers (level C)**  Copeptin, pmol/L (IQR)  IL6, ng/L (IQR)  NFL, ng/L (IQR)  Tau, ng/L (IQR)  GFAP, ng/L (IQR)  UCHL1, ng/L (IQR) | 20.5 (8.6-40.8)  65 (31-141)  53 (30-121)  1 (1-2)  24 (13-37)  161 (107-239) | 46.2 (22.8-89.4)  132 (52-339)  3344 (845-7838)  38 (5-245)  131 (60-353)  684 (279-1582) | <0.001  <0.001  <0.001  <0.001  <0.001  <0.001 | 38.0  38.2  34.3  34.1  34.0  33.9 |

**Table 1D. Variables collected between 48-72 hours of intensive care observation and treatment for good outcome (CPC 1-2) and poor outcome (CPC 3-5) patients after six months**

The variables are grouped into standard ICU observation variables, clinically accessible biomarkers and research-grade biomarkers, all collected during the TTM-trial. Data are presented as *n* (%) or median (IQR). *n* denotes the number of cases with valid data. A *p*-value of <0.05 was considered significant. ICU, Intensive care unit. CPC, Cerebral performance category. IQR, Interquartile range. CRP, C-reactive protein (mg/L). INR, International normalized ratio. EF, Ejection fraction. MAP, Mean arterial pressure. VT, Ventricular tachycardia. VF, Ventricular fibrillation. CPR, Cardiopulmonary Resuscitation. GCS, Glasgow coma scale. BNP, Brain natriuretic peptide. NSE, Neuron-specific enolase. PCT, Procalcitonin. S100B, S100 calcium-binding protein B. TNT, Troponin T. IL6, Interleukin 6. NFL, Neurofilament light. GFAP, Glial fibrillary acidic protein. UCHL1, Ubiquitin carboxy-terminal hydrolase L1.

^a^ Lowest PaO2 (kPa) with the corresponding highest FiO2 (%), PaO_2_ >60 kPa was changed to 60 kPa. ^b^ INR >10 was changed to 10.
